# Supplementary material for: In-silico trial of intracranial flow diverters replicates and expands insights from conventional clinical trials
Source: Nat Commun. 2021 Jun 23;12:3861. doi: 10.1038/s41467-021-23998-w (PMC8222326; doi:10.1038/s41467-021-23998-w)
Supplement: Supplementary file 1 — Supplementary Information [file 41467_2021_23998_MOESM1_ESM.pdf]

In-silico trial of intracranial flow diverters replicates and expands insights from conventional clinical trials: Supplementary material

Ali Sarrami-Foroushani, Toni Lassila, Michael MacRaild, Joshua Asquith, Kit C. B. Roes, James V. Byrne, Alejandro F. Frangi

Supplementary Table 1: Haemodynamic characteristics of aneurysms with/without a side branch.

	Normotensive			Hypertensive		
	**With an SB**	**Without an SB**	**P-Value**	**With an SB**	**Without an SB**	**P-Value**
**Number of aneurysms**	34	48		34	48	
**Sac STAV, pre-FD, m/s**						
**Mean ± SD**	0.18±0.12	0.21±0.14	0.43	0.19±0.11	0.21±0.14	0.44
**Median**	0.15	0.16		0.15	0.17	
**Range**	0.04-0.52	0.03-0.64		0.05-0.49	0.04-0.60	
**Neck MTAV, pre-FD, m/s**						
**Mean ± SD**	0.56±0.25	0.53±0.22	0.49	0.55±0.24	0.50±0.22	0.43
**Median**	0.54	0.50		0.53	0.49	
**Range**	0.16-1.36	0.22-1.15		0.17-1.23	0.21-1.10	
**Sac STAV reduction, %**						
**Mean ± SD**	41.5±18.8	54.1±14.02	<0.001	34.1±15.1	39.2±28.9	0.35
**Median**	43.7	55.6		35.6	44.02	
**Range**	-2.8-78.3	22.9-79.2		0.4-63.5	6.4-69.8	
**Neck MTAV reduction, %**						
**Mean ± SD**	33.9±16.6	45.3±17.2	<0.001	29.4±13.9	35.4±20.3	0.14
**Median**	35.6	50.4		31.8	40.2	
**Range**	-9.3-72.8	4.6-75.8		-7.0-54.5	2.4-60.80	

SB = side branch. STAV = space-and-time-averaged velocity. FD = flow diverter. MTAV = maximum time-averaged velocity.

Supplementary Table 2: Haemodynamic characteristics of large and small size aneurysms.

	Normotensive			Hypertensive		
	**Size>10mm**	**Size<10mm**	**P-Value**	**Size>10mm**	**Size<10mm**	**P-Value**
**Number of aneurysms**	26	56		26	56	
**Sac STAV, pre-FD, m/s**						
**Mean ± SD**	0.14±0.08	0.22±0.14	<0.001	0.15±0.09	0.22±0.14	0.01
**Median**	0.10	0.19		0.12	0.19	
**Range**	0.03-0.34	0.04-0.64		0.04-0.35	0.05-0.60	
**Neck MTAV, pre-FD, m/s**						
**Mean ± SD**	0.51±0.16	0.55±0.26	0.41	0.49±0.16	0.54±0.25	0.43
**Median**	0.48	0.54		0.47	0.53	
**Range**	0.28-1.02	0.16-1.36		0.24-1.00	0.17-1.23	
**Sac STAV reduction, %**						
**Mean ± SD**	45.6±18.2	50.4±16.7	0.25	35.2±15.7	38.0±27.3	0.63
**Median**	46.5	52.2		34.4	42.0	
**Range**	-2.8-79.2	3.9-78.3		0.7-61.5	0.4-69.8	
**Neck MTAV reduction, %**						
**Mean ± SD**	35.9±21.0	42.8±15.8	0.10	29.6±17.2	34.4±18.4	0.27
**Median**	35.4	41.7		31.2	35.9	
**Range**	-9.3-75.8	7.8-72.9		-7.0-59.5	8.6-60.8	

STAV = space-and-time-averaged velocity. FD = flow diverter. MTAV = maximum time-averaged velocity.

Supplementary Table 3: Haemodynamic characteristics of aneurysms with high and low aspect ratios.

	Normotensive			Hypertensive		
	**AR>1.6**	**AR<1.6**	**P-Value**	**AR>1.6**	**AR<1.6mm**	**P-Value**
**Number of aneurysms**	18	64		18	64	
**Sac STAV, pre-FD, m/s**						
**Mean ± SD**	0.09±0.04	0.23±0.13	<0.001	0.10±0.05	0.23±0.13	<0.001
**Median**	0.09	0.19		0.09	0.19	
**Range**	0.03-0.24	0.04-0.64		0.04-0.25	0.05-0.60	
**Neck MTAV, pre-FD, m/s**						
**Mean ± SD**	0.50±0.17	0.55±0.25	0.43	0.48±0.17	0.54±0.24	0.33
**Median**	0.48	0.54		0.47	0.53	
**Range**	0.28-1.02	0.16-1.36		0.24-1.00	0.17-1.23	
**Sac STAV reduction, %**						
**Mean ± SD**	50.9±15.9	48.3±17.6	0.58	37.0±14.9	37.1±26.3	0.98
**Median**	51.8	50.0		36.5	40.7	
**Range**	23.0-79.2	-2.8-78.3		6.4-60.2	0.4-69.8	
**Neck MTAV reduction, %**						
**Mean ± SD**	40.2±20.0	40.7±17.3	0.92	32.2±16.1	33.1±18.7	0.84
**Median**	36.2	41.2		30.7	35.4	
**Range**	4.6-75.8	-9.3-72.8		2.4-59.5	-7.0-60.8	

STAV = space-and-time-averaged velocity. FD = flow diverter. MTAV = maximum time-averaged velocity.

Supplementary Table 4: Haemodynamic characteristics of normotensive and hypertensive aneurysms.

	Normotensive	Hypertensive	P-value
**Number of aneurysms**	82	82	
**Sac STAV, pre-FD, m/s**			
**Mean ± SD**	0.20±0.13	0.20±0.13	0.82
**Median**	0.15	0.16	
**Range**	0.03-0.64	0.04-0.60	
**Neck MTAV, pre-FD, m/s**			
**Mean ± SD**	0.54±0.23	0.52±0.23	0.61
**Median**	0.52	0.50	
**Range**	0.16-1.36	0.17-1.23	
**Sac STAV reduction, %**			
**Mean ± SD**	48.9±17.2	37.1±24.2	<0.001
**Median**	50.0	39.4	
**Range**	-2.8-79.2	0.4-69.8	
**Neck MTAV reduction, %**			
**Mean ± SD**	40.6±17.8	32.9±18.1	0.007
**Median**	40.3	34.0	
**Range**	-9.3-75.8	-7.0-60.8	

STAV = space-and-time-averaged velocity. FD = flow diverter. MTAV = maximum time-averaged velocity.
