# Supplementary material for: In-silico trial of intracranial flow diverters replicates and expands insights from conventional clinical trials
Source: Nat Commun. 2021 Jun 23;12:3861. doi: 10.1038/s41467-021-23998-w (PMC8222326; doi:10.1038/s41467-021-23998-w)
Supplement: Supplementary file 3 — Reporting Summary [file 41467_2021_23998_MOESM3_ESM.pdf]

nature research

Corresponding author(s): Prof. Alejandro F. Frangi

Last updated by author(s): May 17, 2021

Reporting Summary

Nature Research wishes to improve the reproducibility of the work that we publish. This form provides structure for consistency and transparency in reporting. For further information on Nature Research policies, see our Editorial Policies and the Editorial Policy Checklist.

Statistics

For all statistical analyses, confirm that the following items are present in the figure legend, table legend, main text, or Methods section.

n/a	Confirmed	
☐	☒	The exact sample size (n) for each experimental group/condition, given as a discrete number and unit of measurement
☒	☐	A statement on whether measurements were taken from distinct samples or whether the same sample was measured repeatedly
☐	☒	The statistical test(s) used AND whether they are one- or two-sided<br>*Only common tests should be described solely by name; describe more complex techniques in the Methods section.*
☐	☒	A description of all covariates tested
☐	☒	A description of any assumptions or corrections, such as tests of normality and adjustment for multiple comparisons
☐	☒	A full description of the statistical parameters including central tendency (e.g. means) or other basic estimates (e.g. regression coefficient) AND variation (e.g. standard deviation) or associated estimates of uncertainty (e.g. confidence intervals)
☐	☒	For null hypothesis testing, the test statistic (e.g. F, t, r) with confidence intervals, effect sizes, degrees of freedom and P value noted<br>*Give P values as exact values whenever suitable.*
☒	☐	For Bayesian analysis, information on the choice of priors and Markov chain Monte Carlo settings
☒	☐	For hierarchical and complex designs, identification of the appropriate level for tests and full reporting of outcomes
☒	☐	Estimates of effect sizes (e.g. Cohen's d, Pearson's r), indicating how they were calculated

Our web collection on statistics for biologists contains articles on many of the points above.

Software and code

Policy information about availability of computer code

Data collection	All computational models used in this study have been described in previously published works. The digital device implantation model is described in Larrabide et al. 2012. A standard Navier-Stokes equation solver in ANSYS CFX v19.1 (Ansys Inc., Canonsburg, PA, USA) was used to solve the flow problems. The clotting model is described in Sarrami-Foroushani et al. 2019. A reference implementation of the clotting model in ANSYS CFX is available from the authors upon reasonable request.
Data analysis	No custom algorithms or software was used for data analysis.

For manuscripts utilizing custom algorithms or software that are central to the research but not yet described in published literature, software must be made available to editors and reviewers. We strongly encourage code deposition in a community repository (e.g. GitHub). See the Nature Research guidelines for submitting code & software for further information.

Data

Policy information about availability of data

All manuscripts must include a data availability statement. This statement should provide the following information, where applicable:

- Accession codes, unique identifiers, or web links for publicly available datasets
- A list of figures that have associated raw data
- A description of any restrictions on data availability

The imaging data that support the findings of this study are available from the Centre for Computational Imaging & Simulation Technologies in Biomedicine (www.cistib.org) at the University of Leeds as coordinators of the @neurIST Consortium (www.aneurist.org). Restrictions apply to the availability of these data, which were used under a Consortium Agreement for the current study, and so are not publicly available. However, imaging data are available from the authors upon reasonable request and with permission of the @neurIST Consortium.

Field-specific reporting

Please select the one below that is the best fit for your research. If you are not sure, read the appropriate sections before making your selection.

- [x] Life sciences
- [ ] Behavioural & social sciences
- [ ] Ecological, evolutionary & environmental sciences

For a reference copy of the document with all sections, see nature.com/documents/nr-reporting-summary-flat.pdf

Life sciences study design

All studies must disclose on these points even when the disclosure is negative.

Sample size	A power calculation was performed for the superiority of a binary outcome in flow reduction with power 90% and significance level 0.05. The resulting sample size was 65.
Data exclusions	Inclusion/exclusion criteria for FD-PASS followed guidelines of previous clinical trials. The primary cause of exclusion was poor image resolution that prevented the creation of virtual anatomy models amenable to computational simulations. The final FD-PASS cohort consisted of 2 x 82 = 164 virtual patient cases.
Replication	The in-silico trial can be re-run automatically from the previously generated virtual anatomies to reproduce the results.
Randomization	No randomisation was performed as all virtual patients were treated with the same device. Adjustment for covariates was not considered applicable because of the modelling underlying the in silico experiments, as the objective was to compare at population level (uncorrected).
Blinding	No blinding was performed, however the simulation models have been previously validated and the simulation pipeline can be run automatically to produce the outputs in the Source Data.

Reporting for specific materials, systems and methods

We require information from authors about some types of materials, experimental systems and methods used in many studies. Here, indicate whether each material, system or method listed is relevant to your study. If you are not sure if a list item applies to your research, read the appropriate section before selecting a response.

Materials & experimental systems

n/a	Involved in the study	
x		Antibodies
x		Eukaryotic cell lines
x		Palaeontology and archaeology
x		Animals and other organisms
x		Human research participants
x		Clinical data
x		Dual use research of concern

Methods

n/a	Involved in the study	
x		ChIP-seq
x		Flow cytometry
x		MRI-based neuroimaging
